# 2-Cyano­anilinium perchlorate

**DOI:** 10.1107/S1600536810003077

**Published:** 2010-01-30

**Authors:** Li-Jing Cui, Xin-Yuan Chen

**Affiliations:** a College of Chemistry and Chemical Engineering, Southeast University, Nanjing 210096, People’s Republic of China

## Abstract

In the title compound, C_7_H_7_N_2_^+^·ClO_4_^−^, the cation is almost planar (r.m.s. deviation = 0.042 Å). In the crystal structure, the cations and anions are linked into a two-dimensional network parallel to (100) by N—H⋯O hydrogen bonds.

## Related literature

For the crystal structure of 2-cyano­anilinium chloride, see: Oueslati *et al.* (2005 ▶). For Cl—O distances, see: Messai *et al.* (2009 ▶).
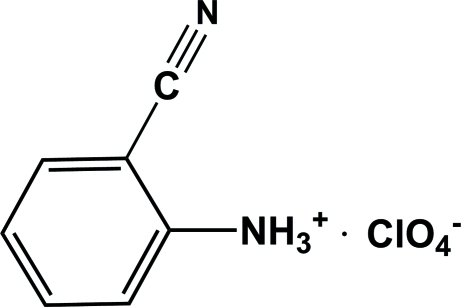


## Experimental

### 

#### Crystal data

C_7_H_7_N_2_^+^·ClO_4_^−^
*M* *_r_* = 218.60
Monoclinic, 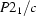

*a* = 11.089 (2) Å
*b* = 7.4561 (15) Å
*c* = 13.872 (5) Å
β = 128.454 (18)°
*V* = 898.2 (4) Å^3^
*Z* = 4
Mo *K*α radiation
μ = 0.42 mm^−1^
*T* = 298 K
0.40 × 0.05 × 0.05 mm

#### Data collection

Rigaku Mercury2 diffractometer
Absorption correction: multi-scan (*CrystalClear*; Rigaku, 2005 ▶) *T*_min_ = 0.90, *T*_max_ = 1.00
9026 measured reflections
2070 independent reflections
1761 reflections with *I* > 2σ(*I*)
*R*_int_ = 0.041

#### Refinement

*R*[*F* ^2^ > 2σ(*F* ^2^)] = 0.044
*wR*(*F* ^2^) = 0.117
*S* = 1.11
2070 reflections
128 parameters
H-atom parameters constrained
Δρ_max_ = 0.32 e Å^−3^
Δρ_min_ = −0.30 e Å^−3^

### 

Data collection: *CrystalClear* (Rigaku, 2005 ▶); cell refinement: *CrystalClear*; data reduction: *CrystalClear*; program(s) used to solve structure: *SHELXS97* (Sheldrick, 2008 ▶); program(s) used to refine structure: *SHELXL97* (Sheldrick, 2008 ▶); molecular graphics: *SHELXTL* (Sheldrick, 2008 ▶); software used to prepare material for publication: *SHELXTL*.

## Supplementary Material

Crystal structure: contains datablocks I, global. DOI: 10.1107/S1600536810003077/ci5023sup1.cif

Structure factors: contains datablocks I. DOI: 10.1107/S1600536810003077/ci5023Isup2.hkl

Additional supplementary materials:  crystallographic information; 3D view; checkCIF report

## Figures and Tables

**Table 1:** Hydrogen-bond geometry (Å, °)

*D*—H⋯*A*	*D*—H	H⋯*A*	*D*⋯*A*	*D*—H⋯*A*
N2—H2*A*⋯O4^i^	0.89	2.14	2.936 (2)	148
N2—H2*B*⋯O4^ii^	0.89	2.24	3.007 (3)	144
N2—H2*C*⋯O1^iii^	0.89	1.98	2.842 (2)	161

Symmetry codes: (i) 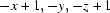; (ii) 
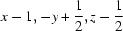
; (iii) 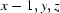.

**Fig. 1.**
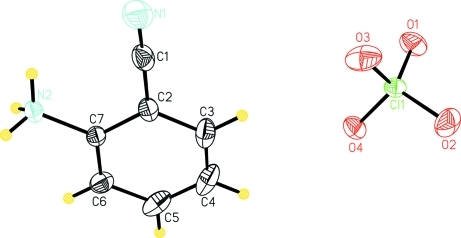
The asymmetric unit of the title compound with the atomic numbering scheme. Displacement ellipsoids were drawn at the 30% probability level.

**Fig. 2.**
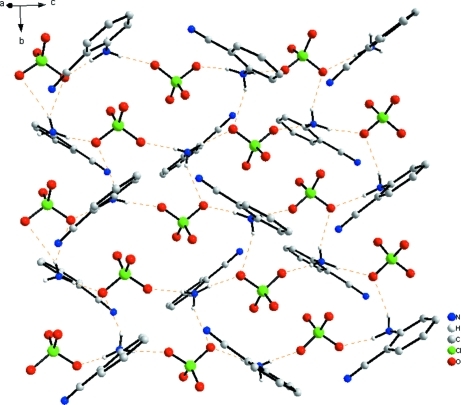
The crystal packing of the title compound, showing a two-dimensional network parallel to the (100). H atoms not involved in hydrogen bonding (dashed line) have been omitted for clarity.

## supplementary crystallographic information

### Comment

Aniline derivatives attracted more attention as phase transition dielectric materials for their applications in micro-electronics and memory storage. With the purpose of obtaining phase transition crystals of 2-aminobenzonitrile salts, its interaction with various acids has been studied and we have obtained a series of new materials with this organic molecule. In this paper, we describe the crystal structure of the title compound, 2-cyanoanilinium perchlorate.

The asymmetric unit is composed of a 2-cyanoanilinium cation and a perchlorate anion (Fig.1). The anion displays a typical tetrahedral geometry around Cl atom and the Cl—O distances compare well with previously reported values (Messai *et al.*, 2009). The cation is almost planar (r.m.s. deviation 0.042 Å; maximum atomic deviation from coplanarity is 0.073 (2) Å by atom N1). The C—NH_3_ [1.466 (2) Å] and C≡N [1.143 (3) Å] distances in the 2-cyanoanilinium cation are longer compared to the corresponding distances in the crystal structure of 2-cyanoanilinium chloride (1.457 (4) Å, 1.137 (4) Å; Oueslati *et al.*, 2005).

In the crystal structure, all the amine group H atoms are involved in N—H···O hydrogen bonds (Table 1). The N—H···O hydrogen bonds link the ionic units into a two-dimensional network parallel to the *ac* plane (Fig. 2).

### Experimental

The commercial 2-aminobenzonitrile (3 mmol, 324 mg) was dissolved in a water-HClO_4_ (50:1 v/v) solution. The solvent was slowly evaporated in air affording colourless crystals of the title compound suitable for X-ray analysis.

While the permittivity measurement shows that there is no phase transition within the temperature range (from 100 K to 400 K), and the permittivity is 7.8 at 1 MHz at room temperature.

### Refinement

All H atoms were initially located in a difference Fourier map. They were then constrained to an ideal geometry, with  C–H = 0.93 Å, N–H = 0.89 Å and *U*_iso_(H) = 1.2U_eq_(C) and 1.5U_eq_(N). A rotating-group model was used for the -NH_3_ group.

### Crystal data

**Table:**

C_7_H_7_N_2_^+^·ClO_4_^−^	*F*(000) = 448
*M**_r_* = 218.60	*D*_x_ = 1.617 Mg m^−^^3^
Monoclinic, *P*2_1_/*c*	Mo *K*α radiation, λ = 0.71073 Å
Hall symbol: -P 2ybc	Cell parameters from 1761 reflections
*a* = 11.089 (2) Å	θ = 3.3–27.5°
*b* = 7.4561 (15) Å	µ = 0.42 mm^−^^1^
*c* = 13.872 (5) Å	*T* = 298 K
β = 128.454 (18)°	Needle, colourless
*V* = 898.2 (4) Å^3^	0.40 × 0.05 × 0.05 mm
*Z* = 4	

### Data collection

**Table:**

Rigaku Mercury2 diffractometer	2070 independent reflections
Radiation source: fine-focus sealed tube	1761 reflections with *I* > 2σ(*I*)
graphite	*R*_int_ = 0.041
Detector resolution: 13.6612 pixels mm^-1^	θ_max_ = 27.5°, θ_min_ = 3.3°
CCD profile fitting scans	*h* = −14→14
Absorption correction: multi-scan (*CrystalClear*; Rigaku, 2005)	*k* = −9→9
*T*_min_ = 0.90, *T*_max_ = 1.00	*l* = −18→18
9026 measured reflections	

### Refinement

**Table:**

Refinement on *F*^2^	Primary atom site location: structure-invariant direct methods
Least-squares matrix: full	Secondary atom site location: difference Fourier map
*R*[*F*^2^ > 2σ(*F*^2^)] = 0.044	Hydrogen site location: inferred from neighbouring sites
*wR*(*F*^2^) = 0.117	H-atom parameters constrained
*S* = 1.11	*w* = 1/[σ^2^(*F*_o_^2^) + (0.0456*P*)^2^ + 0.522*P*] where *P* = (*F*_o_^2^ + 2*F*_c_^2^)/3
2070 reflections	(Δ/σ)_max_ = 0.001
128 parameters	Δρ_max_ = 0.32 e Å^−^^3^
0 restraints	Δρ_min_ = −0.30 e Å^−^^3^

### Special details

**Table:**

Geometry. All esds (except the esd in the dihedral angle between two l.s. planes) are estimated using the full covariance matrix. The cell esds are taken into account individually in the estimation of esds in distances, angles and torsion angles; correlations between esds in cell parameters are only used when they are defined by crystal symmetry. An approximate (isotropic) treatment of cell esds is used for estimating esds involving l.s. planes.
Refinement. Refinement of *F*^2^ against ALL reflections. The weighted *R*-factor wR and goodness of fit *S* are based on *F*^2^, conventional *R*-factors *R* are based on *F*, with *F* set to zero for negative *F*^2^. The threshold expression of *F*^2^ > σ(*F*^2^) is used only for calculating *R*-factors(gt) etc. and is not relevant to the choice of reflections for refinement. *R*-factors based on *F*^2^ are statistically about twice as large as those based on *F*, and *R*- factors based on ALL data will be even larger.

### Fractional atomic coordinates and isotropic or equivalent isotropic displacement parameters (Å^2^)

**Table:**

	*x*	*y*	*z*	*U*_iso_*/*U*_eq_	
N2	0.10356 (18)	0.1622 (2)	0.40630 (16)	0.0351 (4)	
H2A	0.0666	0.0523	0.3784	0.053*	
H2B	0.0743	0.2332	0.3435	0.053*	
H2C	0.0675	0.2053	0.4438	0.053*	
N1	0.2247 (3)	0.4407 (3)	0.2725 (2)	0.0647 (7)	
C7	0.2722 (2)	0.1553 (3)	0.49390 (19)	0.0327 (4)	
C1	0.2853 (3)	0.3504 (3)	0.3573 (2)	0.0472 (6)	
C5	0.5007 (3)	0.0559 (4)	0.6835 (2)	0.0629 (8)	
H5	0.5493	−0.0064	0.7572	0.075*	
C2	0.3588 (2)	0.2429 (3)	0.4665 (2)	0.0386 (5)	
C6	0.3415 (3)	0.0628 (3)	0.6013 (2)	0.0469 (6)	
H6	0.2827	0.0054	0.6189	0.056*	
C4	0.5883 (3)	0.1403 (4)	0.6574 (3)	0.0662 (8)	
H4	0.6952	0.1343	0.7133	0.079*	
C3	0.5183 (3)	0.2326 (4)	0.5496 (3)	0.0547 (7)	
H3	0.5775	0.2886	0.5320	0.066*	
Cl1	0.91401 (6)	0.33466 (7)	0.57188 (4)	0.03477 (17)	
O4	0.8898 (2)	0.2197 (2)	0.64175 (15)	0.0471 (4)	
O3	0.7769 (2)	0.4305 (3)	0.48297 (16)	0.0681 (6)	
O2	1.0336 (2)	0.4577 (2)	0.65476 (18)	0.0610 (5)	
O1	0.9559 (2)	0.2236 (3)	0.51274 (17)	0.0574 (5)	

### Atomic displacement parameters (Å^2^)

**Table:**

	*U*^11^	*U*^22^	*U*^33^	*U*^12^	*U*^13^	*U*^23^
N2	0.0340 (9)	0.0349 (9)	0.0403 (10)	−0.0018 (7)	0.0250 (8)	−0.0023 (7)
N1	0.0733 (16)	0.0653 (15)	0.0617 (14)	−0.0195 (13)	0.0451 (13)	−0.0017 (12)
C7	0.0326 (10)	0.0306 (10)	0.0365 (10)	−0.0049 (8)	0.0223 (9)	−0.0081 (8)
C1	0.0499 (13)	0.0460 (13)	0.0576 (15)	−0.0166 (11)	0.0393 (13)	−0.0111 (12)
C5	0.0518 (15)	0.0571 (16)	0.0415 (13)	0.0008 (13)	0.0101 (12)	−0.0004 (12)
C2	0.0391 (11)	0.0351 (11)	0.0491 (12)	−0.0064 (9)	0.0311 (10)	−0.0094 (9)
C6	0.0476 (13)	0.0474 (13)	0.0384 (12)	−0.0070 (11)	0.0232 (11)	−0.0029 (10)
C4	0.0314 (12)	0.0587 (16)	0.0718 (19)	−0.0030 (12)	0.0140 (13)	−0.0145 (15)
C3	0.0393 (13)	0.0495 (14)	0.0762 (18)	−0.0111 (11)	0.0364 (14)	−0.0166 (13)
Cl1	0.0407 (3)	0.0312 (3)	0.0334 (3)	0.0017 (2)	0.0235 (2)	−0.00068 (19)
O4	0.0561 (10)	0.0436 (9)	0.0532 (10)	−0.0028 (8)	0.0397 (9)	0.0023 (8)
O3	0.0669 (12)	0.0745 (14)	0.0424 (10)	0.0332 (11)	0.0238 (9)	0.0151 (9)
O2	0.0660 (12)	0.0423 (10)	0.0638 (11)	−0.0212 (9)	0.0349 (10)	−0.0126 (8)
O1	0.0724 (12)	0.0587 (11)	0.0635 (11)	0.0041 (9)	0.0534 (11)	−0.0085 (9)

### Geometric parameters (Å, °)

**Table:**

N2—C7	1.466 (2)	C5—H5	0.93
N2—H2A	0.89	C2—C3	1.388 (3)
N2—H2B	0.89	C6—H6	0.93
N2—H2C	0.89	C4—C3	1.367 (4)
N1—C1	1.143 (3)	C4—H4	0.93
C7—C6	1.364 (3)	C3—H3	0.93
C7—C2	1.396 (3)	Cl1—O3	1.4181 (18)
C1—C2	1.437 (4)	Cl1—O2	1.4233 (18)
C5—C4	1.381 (4)	Cl1—O1	1.4315 (17)
C5—C6	1.385 (4)	Cl1—O4	1.4385 (17)
			
C7—N2—H2A	109.5	C7—C6—C5	118.8 (2)
C7—N2—H2B	109.5	C7—C6—H6	120.6
H2A—N2—H2B	109.5	C5—C6—H6	120.6
C7—N2—H2C	109.5	C3—C4—C5	120.2 (2)
H2A—N2—H2C	109.5	C3—C4—H4	119.9
H2B—N2—H2C	109.5	C5—C4—H4	119.9
C6—C7—C2	121.2 (2)	C4—C3—C2	120.0 (2)
C6—C7—N2	118.99 (19)	C4—C3—H3	120.0
C2—C7—N2	119.78 (19)	C2—C3—H3	120.0
N1—C1—C2	177.1 (3)	O3—Cl1—O2	109.53 (13)
C4—C5—C6	120.8 (3)	O3—Cl1—O1	110.35 (12)
C4—C5—H5	119.6	O2—Cl1—O1	111.38 (12)
C6—C5—H5	119.6	O3—Cl1—O4	109.75 (12)
C3—C2—C7	119.0 (2)	O2—Cl1—O4	108.07 (11)
C3—C2—C1	120.1 (2)	O1—Cl1—O4	107.72 (11)
C7—C2—C1	120.8 (2)		
			
C6—C7—C2—C3	1.0 (3)	C4—C5—C6—C7	−0.3 (4)
N2—C7—C2—C3	−179.0 (2)	C6—C5—C4—C3	0.2 (4)
C6—C7—C2—C1	−175.6 (2)	C5—C4—C3—C2	0.5 (4)
N2—C7—C2—C1	4.4 (3)	C7—C2—C3—C4	−1.1 (4)
C2—C7—C6—C5	−0.3 (4)	C1—C2—C3—C4	175.5 (2)
N2—C7—C6—C5	179.7 (2)		

### Hydrogen-bond geometry (Å, °)

**Table:**

*D*—H···*A*	*D*—H	H···*A*	*D*···*A*	*D*—H···*A*
N2—H2A···O4^i^	0.89	2.14	2.936 (2)	148
N2—H2B···O4^ii^	0.89	2.24	3.007 (3)	144
N2—H2C···O1^iii^	0.89	1.98	2.842 (2)	161

Symmetry codes: (i) −*x*+1, −*y*, −*z*+1; (ii) *x*−1, −*y*+1/2, *z*−1/2; (iii) *x*−1, *y*, *z*.
